# Proxalutamide Significantly Accelerates Viral Clearance and Reduces Time to Clinical Remission in Patients with Mild to Moderate COVID-19: Results from a Randomized, Double-Blinded, Placebo-Controlled Trial

**DOI:** 10.7759/cureus.13492

**Published:** 2021-02-22

**Authors:** Flavio A Cadegiani, John McCoy, Carlos Gustavo Wambier, Sergio Vaño-Galván, Jerry Shapiro, Antonella Tosti, Ricardo A Zimerman, Andy Goren

**Affiliations:** 1 Internal Medicine: Diabetes and Endocrinology, Applied Biology Inc, Irvine, USA; 2 Clinical Endocrinology, Federal University of São Paulo, São Paulo, BRA; 3 Research & Development, Applied Biology Inc, Irvine, USA; 4 Dermatology, The Alpert Medical School of Brown University, Providence, USA; 5 Dermatology, Ramon y Cajal Hospital, Madrid, ESP; 6 Ronald O. Perelman Department of Dermatology, New York University, New York, USA; 7 Dermatology, University of Miami Health System, Miami, USA; 8 Infectious Diseases, Hospital da Brigada Militar, Porto Alegre, BRA; 9 Dermatology, Applied Biology Inc, Irvine, USA

**Keywords:** covid-19, sars-cov-2, androgen receptor, androgenetic alopecia, anti-androgen therapy, transmembrane protease serine 2, tmprss2, proxalutamide

## Abstract

Background

The entry of severe acute respiratory syndrome coronavirus 2 (SARS-CoV-2) into type II pneumocytes is dependent on a modification of viral spike proteins by transmembrane protease serine 2 (TMPRSS2) expressed on the surface of human cells. TMPRSS2 is regulated by the androgen receptor, hence, SARS-CoV-2 infectivity is indirectly dependent on androgenic status and phenotype. Previously, we have reported that men affected by androgenetic alopecia (AGA) are overrepresented in severe coronavirus disease 2019 (COVID-19). Additionally, we have reported that men taking antiandrogenic drugs, e.g., 5-alpha-reductase inhibitors (5ARis), are less likely to have severe COVID-19. Here we aimed to test whether the androgen receptor antagonist, Proxalutamide, would be a beneficial treatment for subjects with SARS-CoV-2 infection.

Methods

Male and female subjects were recruited to a double-blinded, randomized, prospective, investigational study of Proxalutamide for the treatment of COVID-19. Mild to moderate, non-hospitalized subjects, who were confirmed positive for SARS-CoV-2, were treated with either Proxalutamide 200 mg/day or placebo. Endpoints for the study were remission time (days) and the percentage of subjects confirmed negative for SARS-CoV-2 on Day 7 after treatment. A negative SARS-CoV-2 test was defined by concentration-time (Ct)>40 determined by real-time reverse transcription-polymerase chain reaction (rtPCR).

Results

Two-hundred thirty-six (2360 subjects were included in the study (108 female, 128 male); 171 were randomized to the Proxalutamide arm and 65 were in the placebo group. On Day 7, SARS-CoV-2 became non-detectable with rtPCR (cT>40) in 82% of the subjects in the Proxalutamide group versus 31% in the placebo group (p < 0.001). The average clinical remission time for patients treated with Proxalutamide was 4.2 ±5.4 days versus 21.8 ±13.0 days in the placebo arm (p < 0.001).

Conclusion

Proxalutamide significantly accelerated viral clearance on Day 7 in mild to moderate COVID-19 patients versus placebo. Further, the time to clinical remission was significantly reduced in patients treated with Proxalutamide versus placebo.

## Introduction

The entry of severe acute respiratory syndrome coronavirus 2 (SARS-CoV-2) into type II pneumocytes proceeds through angiotensin-converting enzyme 2 (ACE2) and is dependent on a modification of a viral spike protein by the transmembrane protease serine 2 (TMPRSS2) expressed on the surface of human cells [1]. The only known regulator of TMPRSS2 is the androgen response element located in the 5’ promoter region of the TMPRSS2 gene [2]. As such, SARS-CoV-2 viral infectivity is likely indirectly regulated by androgens [3-6] and reduction of TMPRSS2 expression by inhibiting the androgen receptor would likely decrease the entry of SARS-CoV-2 into human cells. Previously, we have reported reduced coronavirus disease 2019 (COVID-19) severity in men taking antiandrogens before SARS-CoV-2 infection [7-8]. In addition, genetic variation in the androgen receptor (AR) gene has been correlated with COVID-19 disease severity [9]. Taken together, there is sufficient evidence to suggest that antiandrogen drugs may be a promising treatment for COVID-19.

Proxalutamide (GT0918) is a second-generation androgen receptor antagonist [10-11]. It has a dual mechanism of action in suppressing the AR. In addition to direct AR antagonism, Proxalutamide has been shown to downregulate AR expression, a mechanism that is not present in bicalutamide or enzalutamide [11]. Because of the dual mechanism of action, it is expected to be a more effective and less toxic second-generation anti-androgen drug therapy. Additionally, it has been reported that Proxalutamide lowers the expression of ACE2, which would be beneficial for preventing the entry of SARS-CoV-2 into lung cells.

## Materials and methods

Study design

Potential subjects were recruited to a double-blinded, randomized, placebo-controlled, prospective, investigational study of Proxalutamide treatment of COVID-19. Subjects were recruited through social media and a mailing list from a Brasilia-based Brazilian health care system registry. Subjects with confirmed SARS-CoV-2 infection with mild symptoms (National Institute of Allergy and Infectious Disease (NIAID) COVID-19 eight-point ordinal scale <3), i.e., not requiring admission to a hospital, were randomized to either the Proxalutamide arm (200 mg per day) or the placebo group. All subjects were confirmed SARS-CoV-2 positive by real-time reverse transcription-polymerase chain reaction (rtPCR) testing prior to enrollment. The study was conducted with the approval of the Brazilian National Ethics Committee (Approval number 4.173.074). All patients admitted to the study gave informed consent. All patients, regardless of arm assignment, received standard of care treatment for COVID-19. Baseline characteristics, presence of comorbidities, use of medications, clinical characterization of COVID-19, test results, and disease outcomes were recorded by the principal investigator and managed by the study director.

Study population

Screening of subjects suspected for COVID-19 was conducted by the principal investigator at the study site (Corpometria Institute Brasilia, Brazil). Nasopharyngeal swabs were collected by trained medical personal. SARS-CoV-2 status was laboratory-confirmed rtPCR testing. Subjects were randomized within 24 hours of positive SARS-CoV-2 rtPCR test results. Additional inclusion criteria included (1) 18 years or older; (2) Absence of specific treatments for COVID-19 in the last 72 hours; (3) Absence of contraindications to any of the treatments used in the trial; (4) Oxygen saturation (SatO2) above 92% at the time of the first visit; (5) No signs of complications related to COVID-19, e.g., secondary bacterial infections; and (6) If female, not pregnant, or breastfeeding.

Procedures

Patients were randomized for either Proxalutamide or the placebo arm. Proxalutamide was given 200 mg/day for 15 days or until full COVID-19 remission. For all subjects, nitazoxanide was given 500 mg twice a day after meal for six days and azithromycin was given 500 mg/day one hour before meal for five days, as per one of the standardized therapies suggested by the local Ministry of Health.

Study outcomes

Endpoints for the study were the percentage of subjects confirmed negative for SARS-CoV-2 on Day 7. A negative SARS-CoV-2 test was defined by rtPCR (Automatized Platform, Roche, South San Francisco, CA). A negative SARS-CoV-2 test was defined as a concentration-time (Ct)>40 following the Cobas SARS-CoV-2 rtPCR kit test protocol. A secondary endpoint of average time to clinical remission, i.e., the absence of clinical symptoms, after treatment was also recorded. Time to remission was calculated as the average number of days after randomization in which the patients reported the absence of all COVID-19 symptoms. COVID-19 symptoms included fever, shortness of breath, anosmia, ageusia, cough, runny nose, sore throat, headache, fatigue, muscle or joint pain, weakness, gastrointestinal symptoms, and skin lesions. A secondary analysis was performed for clinical remission, excluding anosmia (loss of smell) and ageusia (loss of taste), as these tend to be the longest-lasting symptoms of COVID-19.

Statistical analysis

The χ2 test for independent proportions was used to perform the analysis of the primary endpoint. Statistical tools were employed to determine statistical significance, which was set at p<0.05. XLSTAT version 2020.3.1.1008 (Addinsoft, Inc., France) was used to perform all statistical analyses.

## Results

Two hundred and thirty-six subjects (236) were included in the trial (108 female, 128 male). One-hundred seventy-one (171) were randomized to the Proxalutamide arm (71 female, 100 male) and 65 (37 female, 28 male) were in the placebo group. A summary of the baseline characteristics of both arms is presented in Table 1.

**Table 1:** Baseline characteristics of the study arms DM: diabetes mellitus; COPD: chronic obstructive pulmonary disorder; ACEi: angiotensin-converting enzyme inhibitors; ARB: angiotensin-2 receptor blockers; CCB: calcium channel blocker; GLP1R: glucagon-like peptide-1 receptor; SGLT2: sodium-glucose cotransporter-2; DPP4: dipeptidyl-peptidase 4; SSRI: selective serotonin reuptake inhibitors

n = 236	Proxalutamide (n = 171)	Placebo (n = 65)	
Baseline characteristics (Mean± SD) Median (IQR)			
Male (M): Female (F)	100M : 71F	28M : 37F	
Age (y/o)	44.5 ± 13.1; 42.5 (17.75)	46.1 ± 12.7; 44.5 (15.75)	
Time-to-treat	4.2 ± 1.8; 4.0 (2.0)	4.0 ± 1.6; 4.0 (2.0)	
Comorbidities, Number (%)			
Obesity	28 (15.7%)	9 (13.6%)	
Hypertension	39 (22.8%)	14 (21.5%)	
Myocardial infarction	2 (1.2%)	0	
Stroke	2 (1.2%)	0	
Congestive heart failure	1 (0.6%)	1 (1.5%)	
Lipid disorders	30 (17.5%)	16 (24.6%)	
DM (type 1 and 2)	15 (8.8%)	6 (9.2%)	
Prediabetes	18 (10.5%)	5 (7.7%)	
Dysglycemia	33 (19.3%)	11 (16.9%)	
Asthma	8 (4.7%)	4 (6.2%)	
COPD	0	0	
Chronic kidney disease	1 (0.6%)	0	
Liver fibrosis/cirrhosis	0	0	
Clinical depression	11 (6.4%)	3 (4.6%)	
Anxiety	29 (16.9%)	7 (10.8%)	
Hypothyroidism	18 (10.5%)	7 (10.8%)	
Autoimmune disorders	1 (0.6%)	0	
Cancer	3 (1.8%)	1 (1.5%)	
Drugs (Number and %)			
Beta-blocker	7 (4.1%)	3 (4.6%)	
ACEi	1 (0.6%)	0	
ARB	25 (14.6%)	5 (7.7%)	
Loop diuretics	2 (1.2%)	0	
Thiazide diuretics	7 (4.1%)	2 (3.1%)	
CCB	15 (8.8%)	3 (4.6%)	
Statins	13 (7.6%)	4 (6.2%)	
Others	2 (1.2%)	0	
Aspirin	5 (2.9%)	1 (1.5%)	
Clopidogrel	0	1 (1.5%)	
Warfarin	0	0	
Xa factor inhibitors	1 (0.6%)	0	
Direct thrombin inhibitors	0	0	
Heparin	0	0	
Metformin	16 (9.4%)	5 (7.7%)	
GLP1R analogs	10 (5.8%)	3 (4.6%)	
SGLT2 inhibitors	16 (9.4%)	3 (4.6%)	
DPP4 inhibitors	2 (1.2%)	1 (1.5%)	
Sulfonylureas	3 (1.8%)	0	
Glitazones	0	0	
Acarbose	0	0	
Insulin	2 (1.2%)	0	
Orlistat	3 (1.8%)	2 (4.5%)	
Levothyroxine	18 (10.1%)	7 (10.6%)	
Hypnotics	6 (3.5%)	1 (1.5%)	
SSRI	17 (9.9%)	4 (6.2%)	
Other antidepressants and mood stabilizers	8 (4.7%)	3 (4.6%)	
Benzodiazepines	4 (2.3%)	0	
Atypical antipsychotics	8 (4.7%)	2 (3.1%)	
Omega-3	3 (1.7%)	0	
Vitamin D	20 (11.7%)	5 (7.7%)	
Zinc	9 (5.3%)	2 (3.1%)	
Vitamin C	14 (8.2%)	5 (7.7%)	
Multivitamin	12 (7.0%)	4 (6.2%)	

On Day 7, the SARS-CoV-2 virus was undetectable in 82% of the subjects in the Proxalutamide group versus 31% in the placebo group (p < 0.0001). The difference in proportions was 51% (95%CI: 42.5% to 66.8%, p<0.0001). The χ2 test for independent proportions was 70.290. Results from the rtPCR findings are presented in Table 2.

**Table 2:** Day 7 COVID-19 status (rtPCR) of patients treated with Proxalutamide or placebo rtPCR: real-time reverse transcription-polymerase chain reaction

	Proxalutamide (n = 171)	Placebo (n = 65)	p-value
COVID-19 rtPCR (Ct>40) negative at Day 7			
Total population	140/171 (82%)	20/65 (31%)	< .001
Men	81/100 (81%)	6/28 (21%)	< .001
Women	60/71 (85%)	14/37 (38%)	< .001

A sub-analysis of the women showed that 85% (60/71) in the Proxalutamide group tested negative after seven days versus 38% (14/37) in the placebo arm. The difference in proportions was 47% (95%CI: 28% to 62.3%, p<0.0001). The χ2 test for independent proportions was 24.843. For the men, 81% (81/100) in the Proxalutamide group tested negative after seven days versus 21% (6/28) in the placebo arm. The difference in proportions was 60% (95%CI: 39.9% to 72.8%, p<0.0001). The χ2 test for independent proportions was 35.834.

The average time to clinical remission in the Proxalutamide group was 4.2 ±5.4 days versus 21.8 ±13.0 days in the placebo arm (p < 0.001). By Day 7 after treatment, 82.5% of subjects had fully recovered (i.e., did not report any symptoms of COVID-19 (see Methods section) versus 24.4% in the placebo arm (p<0.001). A summary of clinical remission times is presented in Table 3. Subjects in the Proxalutamide arm, more frequently reported gastrointestinal treatment-emergent adverse events (TEAE) than subjects in the placebo group, however, no subjects in either arm discontinued the study due to TEAE. A summary of TEAEs is presented in Table 4.

**Table 3:** Average time in days to clinical remission of the Proxalutamide and placebo arms

	Proxalutamide (n = 171)	Placebo (n = 65)	p-value
Time to clinical remission (Days), Mean (±SD)			
Clinical remission minus loss of taste or smell	1.8 (±2.9)	12.0 (±11.7)	< .001
Clinical remission including loss of taste or smell	4.2 (±5.4)	21.8 (±13.0)	< .001
Number of subjects achieving clinical remission at Day 7, No./Total (%)			
Clinical remission minus loss of taste or smell	164/171 (95.9%)	32/66 (48.5%)	< .001
Clinical remission including loss of taste or smell	141/171 (82.5%)	16/66 (24.2%)	< .001

**Table 4:** Treatment-emergent adverse events (TEAE) of the study arms

	Proxalutamide	Placebo
	(n = 171)	(n = 65)
Gastrointestinal		
Diarrhea	39	13
Nausea	28	9
Abdominal pain	19	5
Vomiting	4	1
Abdominal discomfort	17	4
Dyspepsia	20	7
Heartburn	11	6
General		
Fatigue	6	8
Fever	1	5
Disease progression	3	7
Cardiac		
Tachycardia	6	3
Nervous system		
Headache	5	6
Ageusia	0	8
Anosmia	1	10
Musculoskeletal and cognitive tissue		
Arthralgia	0	1
Back pain	3	2
Pain in extremity	2	4
Respiratory, thoracic, and mediastinal		
Shortness of breath	5	4
Total TEAE	170 in 171 subjects	99 in 65 subjects

## Discussion

Effective therapies for COVID-19 are critical while vaccines are being implemented. To date, only a limited amount of studies have been conducted in early COVID-19 subjects. The majority of larger clinical trials were performed in hospitalized patients. Here, we present a randomized controlled double-blinded interventional study of Proxalutamide for the treatment of early COVID-19. Baseline characteristics and comorbidity prevalence were similar between the Proxalutamide arm and the placebo group, and all patients received standard of care treatment for COVID-19, i.e., Proxalutamide or placebo was administered as add-on therapy. Add-on therapies tend to demonstrate fewer results than mono-therapies, particularly for COVID-19, in which there has been some evidence of effectiveness for emerging treatments, e.g., nitazoxanide [12]. However, here we demonstrated statistically significant results.

Viral clearance, as determined by a negative rtPCR SARS-CoV-2 (Ct>40), was enhanced in the Proxalutamide treatment group. Since detected viral ribonucleic acid (RNA) may not necessarily reflect the presence of a viable and live virus, a negative result after initial confirmation of COVID-19 has a high probability of indicating complete recovery. In the study, we observed the percentage of negative rtPCR on Day 7 in the placebo group was slightly higher than previous reports in the literature in untreated patients [13]. Nevertheless, almost three times as many subjects in the Proxalutamide group compared to those from the placebo group tested negative for SARS-CoV-2 on Day 7. Compared to other treatments with in-vitro anti-SARS-CoV-2 activity or preliminary clinical data, to date, no other drug has demonstrated the ability to enhance viral clearance to the extent observed for Proxalutamide [12,14]. Similarly, time to clinical remission was vastly improved in the subjects given Proxalutamide versus the placebo. We observed a five-fold decrease in the average number of days required to be free of symptoms of COVID-19 in the Proxalutamide group versus the placebo. On Day 7, more than three times as many patients had fully recovered from COVID-19 in the Proxalutamide arm as compared to the placebo group.

The significant improvements observed in clinical and virological responses reinforce the efficacy of Proxalutamide for COVID-19. Proxalutamide has demonstrated to be an effective AR suppressor. In addition, Proxalutamide has been demonstrated to downregulate AR expression [10-11]. Finally, the suppression of membrane-attached ACE2 has been described as an additional pleiotropic mechanism of action of Proxalutamide for blocking SARS-CoV-2 cell entry. The collative actions of Proxalutamide make it ideally suited for treating COVID-19. It is possible that additional mechanisms of actions not related to the anti-androgen activity of Proxalutamide are present and have yet to be elucidated, e.g., anti-inflammatory, anti-thrombotic, or other non-androgen mediated anti-viral effects may be present. The expected suppression time for both transmembrane serine protease 2 (TMPRSS-2) and ACE2 after the administration of Proxalutamide does not explain the effects observed clinically in treated patients. We would expect a greater lag between the initiation of treatment and clinical benefit. While further mechanisms of action are yet to be clarified, the substantial differences in the treatment and placebo groups provide convincing evidence for the benefits of treatment with Proxalutamide for mild-to-moderate COVID-19 patients.

## Conclusions

In a randomized, double-blinded, placebo-controlled interventional study in males and females, treatment with Proxalutamide increased SARS-CoV-2 viral clearance at Day 7 and reduced the average number of days to COVID-19 clinical remission when compared to placebo.
